# Direct detection of diverse metabolic changes in virally transformed and Tax-expressing cells by mass spectrometry

**DOI:** 10.1186/1742-4690-8-S1-A179

**Published:** 2011-06-06

**Authors:** Prabhakar Sripadi, Bindesh Shrestha, Rebecca Easley, Lawrence Carpio, Kylene Kehn-Hall, Sebastien Chevalier, Renaud Mahieux, Akos Vertes, Fatah Kashanchi

**Affiliations:** 1 The George Washington University, Department of Chemistry, W. M. Keck Institute of Proteomics Technology and Applications, Washington, DC, 20037, USA; 2 George Mason University, Department of Molecular and Microbiology, National Center for Biodefense and Infectious Diseases, Manassas, VA, 20110, USA; 3 National Institutes of Health/National Cancer Institute, Laboratory of Cellular Oncology, Bethesda, Maryland, 20892, USA; 4 Ecole Normale Supérieure de Lyon, Equipe Oncogenèse Rétrovirale, U758 INSERM, Lyon, France; 5 The George Washington University Medical Center, Department of Microbiology, Immunology, and Tropical Medicine, Washington, DC, 20037, USA

## 

HTLV-1- induced transformation causes extensive changes at the gene, protein and metabolite levels. These changes are usually followed by gene-expression profiling and proteomic analysis. Exploring the metabolic consequences of viral transformation adds to the picture because the viruses rely on the metabolic network of their cellular hosts for survival and replication. Metabolites are small molecules of diverse physico-chemical properties with greatly different abundance levels that make their analysis challenging. Typically optical (e.g., Fourier transform infrared spectrometry), nuclear magnetic resonance (NMR) and mass spectrometric techniques in combination with separation techniques, such as gas chromatography, high performance liquid chromatography (HPLC) and capillary electrophoresis, have been used for metabolomic studies. Here we utilized a new and novel method called laser ablation electrospray ionization (LAESI) to detect metabolites without any processing of samples. When using the LAESI technique to identify metabolic changes in HTLV1 and Tax1 transformed T lymphocytes and in HTLV3 and Tax3 cells, we found virus type specific (HTLV1 vs. HTLV3), expression specific (Tax1 vs. Tax3) and cell type specific (T lymphocytes vs. kidney epithelial cells) changes in the metabolite profiles. We have identified a number of metabolites that are known in the literature to be deregulated in the viral transformation process (e. g., arginine, cAMP, glutathione) as well as multiple novel metabolites that may have implications in HTLV1-induced transformation (e. g., putrescine, N-acetyl aspartic acid, methoxytyramine). These new findings point to metabolic pathways that have a heretofore unexplored role in the viral transformation of host cells.

